# Competing for congestible goods: experimental evidence on parking choice

**DOI:** 10.1038/s41598-020-77711-w

**Published:** 2020-11-30

**Authors:** María Pereda, Juan Ozaita, Ioannis Stavrakakis, Angel Sánchez

**Affiliations:** 1 Grupo de Investigación Ingeniería de Organización y Logística (IOL), Departamento Ingeniería de Organización, Administración de empresas y Estadística, Escuela Técnica Superior de Ingenieros Industriales, Universidad Politécnica de Madrid, C/ José Gutiérrez Abascal, 2, 28006 Madrid, Spain; 2 Unidad Mixta Interdisciplinar de Comportamiento y Complejidad Social (UMICCS) UC3M-UV-UZ, 28911, Leganés, Madrid Spain; 3 Grupo Interdisciplinar de Sistemas Complejos, Departamento de Matemáticas, Universidad Carlos III de Madrid, 28911 Leganés, Madrid Spain; 4 Department of Informatics and Telecommunications, National and Kapodistrian University of Athens, Athens, Greece; 5 Institute UC3M-Santander for Big Data (IBiDat), Universidad Carlos III de Madrid, 28903 Getafe, Madrid Spain; 6 Instituto de Biocomputación y Física de Sistemas Complejos (BIFI), Universidad de Zaragoza, 50018 Zaragoza, Spain

**Keywords:** Applied mathematics, Statistical physics, thermodynamics and nonlinear dynamics, Computational science

## Abstract

Congestible goods describe situations in which a group of people share or use a public good that becomes congested or overexploited when demand is low. We study experimentally a congestible goods problem of relevance for parking design, namely how people choose between a convenient parking lot with few spots and a less convenient one with unlimited space. We find that the Nash equilibrium predicts reasonably well the competition for the convenient parking when it has few spots, but not when it has more availability. We then show that the Rosenthal equilibrium, a bounded-rational approach, is a better description of the experimental results accounting for the randomness in the decision process. We introduce a dynamical model that shows how Rosenthal equilibria can be approached in a few rounds of the game. Our results give insights on how to deal with parking problems such as the design of parking lots in central locations in cities and open the way to better understand similar congestible goods problems in other contexts.

## Introduction

Goods or services can be classified, generally speaking, in terms of their excludability and rivalry^1^. Thus, goods are called excludable if it is possible to prevent people (consumers) who have not paid for it from having access to it, whereas rivalry indicates that one person’s consumption of a product reduces the amount available for consumption by others^2^. Non-excludable goods, i.e., goods or services that can be accessed by anybody without restrictions, describe a wide spectrum of technological, economic or social situations. Often, such situations are in between the usual public goods, arising when there is no rivalry^3^, and common-pool resources^4^, that are subjected to the well-known “tragedy of the commons”^5^. In fact, some goods, called congestible or impure, act as public goods when demand is low, and like common-pool resources when crowded^6^. Well-known examples are education or health, but one could think of many more such as child or elderly care, family services, motorways, public museums, and even intellectual property and wireless networks^7–11^.

A prominent example of a congestible goods is parking space. This is a very important problem in the twentifirst century: for example, cruising for parking spaces accounted for 30% of the traffic congestion in cities on average^12^ generating a correspondingly disproportionate contribution to pollution and greenhouse gases^13^. In view of this and related issues, a lot of effort is being devoted to study parking behavior, and the conclusions are likely to dramatically reduce the number of parking spots^14^. Thus, parking in the center of large cities becomes an impure public goods, and typically it becomes a congested resource having only accommodated a small fraction of the incoming cars. In order to manage parking efficiently, several approaches have been proposed to leverage the availability of smart devices to real-time information on parking availability^15,16^, such as data access via a peer-to-peer (P2P) environment^17,18^, open space detection coupled with information sharing^19^, and networking for data sharing^20^. These and other new technologies provide real-time allocation of slots to demanding drivers and can reduce considerably the waste of searching for parking spaces, although they are of course not problem-free^21^.

A complementary approach to solve this problem (that could provide, for example, guidelines as to how to design effective parking facilities) is to achieve a better understanding of the decision-making process of drivers trying to park in possibly congested lots. This, in turn, leads to consider parking as a strategic decision in the framework of game theory^22,23^. Several works have addressed this question by focusing on the possible heuristics used by drivers^24^ or on recommending them ones to use^25,26^. Models of decision making under bounded rationality have also proven very useful in this context^27^. However, to the best of our knowledge, this question has not been considered from the experimental viewpoint by studying how actual people make their decisions^28^. This paper is intended to fill in this knowledge gap and support the design of further mechanisms of parking management.

To this end, we have designed a parking experiment in which players have to choose between two types of car park areas: one which is cheap or convenient (e.g., because it is located downtown), but has a limited number of spots, and one which is more expensive or less convenient (following the same example, because it is further from downtown) but can accommodate all the demand for parking. When one tries the cheap parking first and fails to find a spot, then there is an extra cost arising from the time wasted or the extra distance driven. As we will see below, depending on the parameters (number of spots, number of cars, costs) the prediction from the Nash equilibrium is that all or a fraction of the participants should try the cheap parking. We are therefore asking here whether this prediction will hold and what can be learned from the actual behavior of people in order to plan the distribution of car park areas in cities. The presentation of our results is organized as follows: we begin by introducing the details of our experimental setup and the corresponding Nash equilibria. Subsequently, we report the results of our experiments and our main findings. We then introduce a theoretical analysis based on the Rosenthal equilibrium^29^ that incorporates a decision model allowing for deviations from the fully rational case assumed under the Nash equilibrium. In addition, we introduce a complementary agent-based model based on reinforcement learning dynamics that sheds light on how the equilibrium may be reached^30^. We conclude by discussing the experimental results in the light of these two approaches and draw lessons for parking management and for applying our ideas to similar situations described as congestible goods problems.

## Results

### Experimental setup

Participants played in groups of $N=20$ subjects. They were informed that they were participating with other 19 people. They played four repetitions of the experiment, one after the other, for different parameterizations of the experiment (see “Methods” section for a detailed description of the experimental setup). In each repetition, the participants had to make the same decision ten times, one per experimental round. If a decision was not made within the 30 s available, the software would decide at random for the participant, and this decision would be marked as “automatic”. Participants were only paid if they had made at least 70% of the decisions.

Participants were supposed to have to park their car, and so they had to decide between two types of car park areas: a yellow parking lot with only $S\in \{5, 10\}$ slots available, or a blue parking lot with an unlimited number of slots. The availability of slots in the yellow parking lot was subject to the demand in each round. If more slots were demanded than those available, they were assigned to the participants that demanded them at random, and the ones that did not get a slot had to proceed to the blue parking lot.

For each decision, the participants were given 100 points at the beginning of the round. They had 30 s to make a decision. The cost of the yellow parking lot was $Q_{{\mathrm{cheap}}} =$ 10 points and the cost of the blue parking lot was $Q_{\mathrm{exp}}\in \{20, 40\}$. In addition, if the decision was to go for the yellow parking lot but there were no free slots available, then the car had to go to the blue parking lot, incurring a cost $Q_{\mathrm{add}}\in \{10, 50\}$ to be added to the cost of the parking $Q_{\mathrm{exp}}$. The possible combinations of the values for the three parameters *S*, $Q_{{\mathrm{cheap}}}$ and $Q_{\mathrm{add}}$ give rise to eight different treatments, which are described in detail in the “Methods” section.

Based on their decision, at the end of each round, the participants had an associated payoff, resulting from subtracting their decision costs from their initial endowed 100 points. After each round, the participants were reminded about their previous decision and informed about the car park they parked, their payoff for that round, and the number of people that actually found a slot in the yellow parking. At the end of the experiment, the participants answered a risk assessment test, as in Ref.^31^, which can be found in the “Supplementary Information” accompanying this article.

### Nash equilibria

The analysis of this type of game for fully rational behaviors was carried out in Ref.^32^. We will not repeat all the details here, but limit ourselves to particularizing the results obtained there for our choice of parameters. When doing this, we find that the number of players who compete for a yellow parking slot at the Nash equilibrium is given by

1 $$\begin{aligned} N^{\mathrm{Nash}} = \left\{ \begin{array}{lll} N &{} {\mathrm{if}} &{} N \le S\left( 1 + \dfrac{Q_{\mathrm{exp}}-Q_{{\mathrm{cheap}}}}{Q_{\mathrm{add}}}\right) , \\ \\ S\left( 1 + \dfrac{Q_{\mathrm{exp}}-Q_{{\mathrm{cheap}}}}{Q_{\mathrm{add}}}\right) &{} {\mathrm{if}} &{} N > S\left( 1 + \dfrac{Q_{\mathrm{exp}}-Q_{{\mathrm{cheap}}}}{Q_{\mathrm{add}}}\right) . \\ \end{array} \right. \end{aligned}$$

This result means that if the number of spots available in the yellow parking lot is small (actually, less than a certain bound that depends on the costs), an equal number of people should compete for those, obtaining them; when the number of spots is larger, then the fraction of competitors does depend on the costs as indicated in Eq. (1). This, in turn, translates into the fact that the probability that a given player decides to compete for a cheap yellow parking slot under the corresponding mixed-action formulation of the game is given by:

2 $$\begin{aligned} p_{{\mathrm{cheap}}}^{\mathrm{Nash}} = \left\{ \begin{array}{lll} 1 &{} {\mathrm{if}} &{} N\le S\left( 1 + \dfrac{Q_{\mathrm{exp}}-Q_{{\mathrm{cheap}}}}{Q_{\mathrm{add}}}\right) , \\ \\ \dfrac{S}{N} \left( 1 + \dfrac{Q_{\mathrm{exp}}-Q_{{\mathrm{cheap}}}}{Q_{\mathrm{add}}}\right) &{} {\mathrm{if}} &{} N > S\left( 1 + \dfrac{Q_{\mathrm{exp}}-Q_{{\mathrm{cheap}}}}{Q_{\mathrm{add}}}\right) . \\ \end{array} \right. \end{aligned}$$

### Experimental results analysis

The analysis was performed by considering the decisions of participants that completed the experiment, and for those, only participant-made decisions (random decisions made by the software were replaced by real people decisions selected at random from the same treatment and round). Due to the dropout of participants during the experiments, some of the groups ended with less than 20 participants. For the analysis, incomplete groups were completed up to 20 participants by sampling from the participant decisions under the same parameterizations of the experiment. Hence, results show only participant-made decisions. The results of these participant-made decisions are comparable to those including automatic random decisions and decisions of participants that did not complete the experiment. These “raw-data” results are shown at Supplementary Figures S1 and S2, together with the average percentage of random decisions per round and treatment (Supplementary Figure S3). Differences between the two sets of results are negligible.

To carry out the analysis, we use the following variables: *decision* is equal to 1 if a player chooses to compete for the yellow parking lot, and 0 if the player chooses blue parking lot. As explained below in the “Methods” section, we will refer to each treatment as $EXP\_S/N\_Q_{\mathrm{exp}}\_Q_{\mathrm{add}}$, i. e., $EXP\_0.5\_20\_10$ means $S=10$, $Q_{\mathrm{exp}}=20$, $Q_{\mathrm{add}}=10$. Each experimental treatment was replicated six times, by six different groups of people.

**Figure 1:**
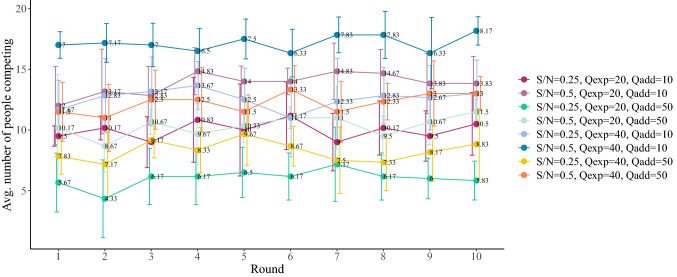
Average number of people competing as a function of round number in the eight treatments of our experiment. Error bars represent ± one standard deviation of the mean.

The first set of analyses examine the impact of the parameters of the experiment on the average number of participants competing for the yellow parking lot during the rounds of the experiment, that is, trying to park in the limited but cheap car park lot. For each round, we compute the average number of people choosing the yellow parking lot in the six repetitions of the treatments. Figure 1 presents this average as a function of the rounds. If we focus on the different *S*/*N* ratios, i.e. the ratio of yellow parking lot spaces available *S* to the number of participants *N*, we can see that competition is higher, meaning that there is more demand for the yellow parking lot, when there are more spaces available and so *S*/*N* is higher. For the same *S*/*N* ratio, the higher $Q_{\mathrm{add}}$, i.e., the cost of competing and having to move eventually to the blue parking lot, the fewer people compete for the yellow parking lot. For the same *S*/*N* ratio and $Q_{\mathrm{add}}$, we see that people compete for the yellow parking lot more if the cost of the blue parking lot is higher. Overall, for the duration of each set of 10 rounds for any given set of parameters, the average number of people competing for each treatment remains constant or with very small variability.

**Figure 2:**
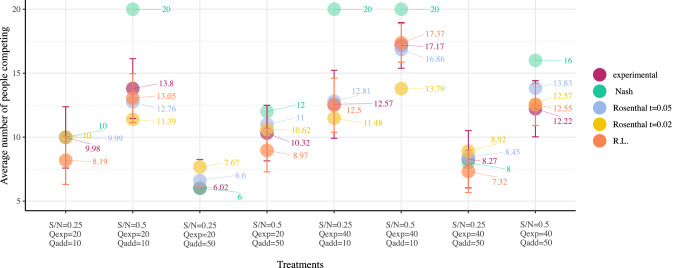
Average number of people competing per treatment versus Nash and Rosenthal equilibria. Magenta: experimental results; turquoise: Nash; blue: Rosenthal, $t=0.05$; yellow: Rosenthal, $t=0.02$; orange: Reinforcement learning (R.L.) model. Error bars represent ± one standard deviation of the mean. Points overlap for all sets on the first treatment, except for R.L. simulation results.

If we focus on the participants decisions after the first five rounds, i.e, in rounds 6–10, all participants chose the same option in at least four of those five rounds. Averaging over all treatments, 58% of the participants chose the competitive option of the yellow parking lot. Turning now to compare the average number of people competing per treatment with Nash equilibria predictions, we compute the average decision per person in all rounds, and then per group of *N* people. This group means are averaged per treatment and shown in Fig. 2 together with the standard deviation of these averages as error bars. Only three of the eight experimental treatments behave as foreseen by the Nash equilibrium (treatments $EXP\_0.25\_20\_10$, $EXP\_0.25\_20\_50$, and $EXP\_0.25\_40\_50$), that is when the Nash equilibrium suggests that less than or equal to half of the participants should compete. When the Nash equilibrium is very large, meaning that all participants should compete, we observe that a number of participants shy away from this competition, and the Nash equilibrium overestimates the actual number of subjects going to the yellow parking lot. In some cases, mostly when the additional cost is low, the discrepancy is quite big.

**Figure 3:**
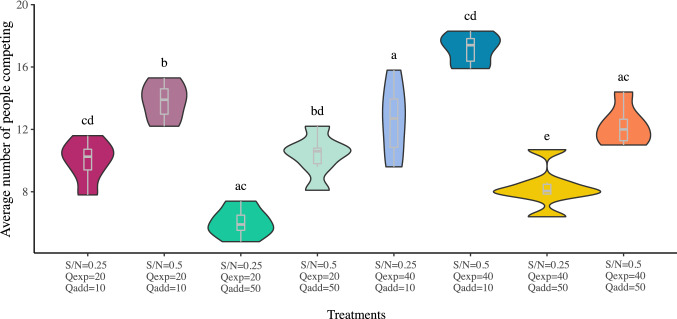
Violin plots and boxplots for the number of people competing per treatment.

In order to assess whether the averages of people competing in the treatments are statistically different, we performed firstly a one-way ANOVA test, and secondly multiple pairwise-comparisons with the Tukey’s range test. The ANOVA test concluded that there are significant differences between the treatments (p-value = $2.99{\text {e}}-15$). The Tukey’s range test determines if two means are different by at least one standard error of the mean. Figure 3 shows the violin plots and boxplots of the number of people competing per treatment and summarize the Tukey test by using letters. The letters on top of the violin plots represent experimental treatments which are not significantly different. For instance, the leftmost condition, labelled ‘cd’, is statistically indistinguishable from all other conditions with which it shares a letter, i.e., from the third, the fourth, the sixth and the eighth treatments. Treatments that are not different may be suggesting that subjects are perceiving them as similar situations. For instance, all treatments with a high cost of failure ($Q_{\mathrm{add}}=50$) are comparable to some extent. Another interesting observation that arises from Fig. 3 is that when the observed behavior is far from the predictions of Nash equilibria, the individual results show larger dispersion, indicated by an almost rectangular shape of the violin. On the contrary, when the fraction of competing people is close to the Nash prediction, we see that individual results tend to cluster around the mean and the prediction. This suggests that the reason why the Nash prediction is not accurate is connected to people having difficulties in estimating what would be a rational response for those choices of parameters.

Finally, as indicated above, we also collected data on the risk aversion of the participants, in order to study whether it is related to their competitive behavior. Strikingly, we find no correlation whatsoever of their risk profile with their average competitive behavior (average number of times they choose the yellow parking lot).

### Rosenthal model

In this section, we consider a theoretical alternative to the predictions of Nash equilibria, namely the Rosenthal model^29^. This is a parametric probabilistic choice model (with one parameter) aiming to capture any unobserved and omitted elements, estimation/computational errors, individual’s mood, perceptual variations or cognitive biases driving human decisions, in line with the fact that individuals are more likely to make better choices than worse choices, but do not necessarily make the very best choices. According to this model, the difference in probabilities with which two actions are played (select a yellow or blue parking slot here) equals a parameter *t* multiplied by the difference of the corresponding expected costs. The parameter *t*, $0\le t\le \infty$ is referred to as the rationality parameter. The case of $t=\infty$ corresponds to decision making under full rationality and for that choice of *t* the corresponding Rosenthal equilibrium coincides with the Nash equilibrium. On the other hand, the case of $t=0$ corresponds to pure random choice. The Rosenthal probabilistic decision model is given by

3 $$\begin{aligned} p_{{\mathrm{cheap}}} - p_{\mathrm{exp}} = t \left( C_{{\mathrm{cheap}}} (p_{{\mathrm{cheap}}} ) - Q_{\mathrm{exp}} \right) , \end{aligned}$$

where $p_{{\mathrm{cheap}}}$ and $p_{\mathrm{exp}}$ are the probabilities for selecting a yellow (cheap) or a blue (expensive) parking slot and $C_{{\mathrm{cheap}}} (p_{{\mathrm{cheap}}}$ is the cost of a player choosing yellow. From the analysis and expressions presented in Ref.^27^ we obtain the following expression for $C_{{\mathrm{cheap}}} (p_{{\mathrm{cheap}}})$:

4 $$\begin{aligned} C_{{\mathrm{cheap}}} (p_{{\mathrm{cheap}}}) = \sum _{k=0}^{N-1} g_{{\mathrm{cheap}}}(k+1) B(k,N-1,p_{{\mathrm{cheap}}}), \end{aligned}$$

where $g_{{\mathrm{cheap}}}(k)$ is the average cost of a player choosing yellow when $k>S$ compete, given by

5 $$\begin{aligned} g_{{\mathrm{cheap}}}(k) = min \left( 1, S/N \right) Q_{{\mathrm{cheap}}} + \left( 1-min \left( 1,S/N\right) \right) \left( Q_{\mathrm{exp}} + Q_{\mathrm{add}}\right) , \, 1\le k \le N \end{aligned}$$

and $B(k,N-1,p_{{\mathrm{cheap}}})$ is the Binomial distribution for k successes in ${\text{ N }}-1$ trials with probability of success per trial equal to $p_{{\mathrm{cheap}}}$.

By solving Eq. (3) for $p_{{\mathrm{cheap}}}$ (under the constraint $p_{{\mathrm{cheap}}} + p_{\mathrm{exp}} = 1$) the Rosenthal equilibrium value $p_{{\mathrm{cheap}}}^{RE}$ is derived for a given rationality parameter *t*. The corresponding results show that the Rosenthal model for $t=0.05$ yields a value for the equilibrium that produces decisions that are closer to the experiments as measured by adding up all the distances from the predicted points to the experimental value (4.66). For $t=\infty$, i.e., the Nash equilibrium, this value is 22.23. A comparison is depicted in Fig. 2. Thus, we can claim that the players in our game do not behave as fully rational players. That is, they do not try to maximize their own utility assuming all players play the same, but they exhibit bounded rationality, as captured by the factor $t=0.05$ that shapes the difference in the probabilities of the two choices by weighing only modestly (small value for *t*) the difference in costs between the two choices they have. On the contrary, the fully rational model would weigh infinitely that cost difference.

Notice that the random choice case gives a large distance from the experimental (21.07), suggesting that the players in our game do not behave as absolutely random players (i.e., they do not select each of the two choices with the same probability of 0.5). This suggests that although there are no suggested values for the rationality parameter *t* to be used, the value of $t=0.05$ for our decision problem leads to decisions that are clearly away from the completely random decisions ($t=0$).

Optimally, exactly $\min (S,N)$ players should compete for the yellow parking slots, so that no player pays $Q_{\mathrm{add}}$ and no player pays $Q_{\mathrm{exp}}$ if there are yellow parking slots available. Clearly, the optimal decision requires coordination and collaboration among the players and is not feasible in the uncoordinated environment we consider here. Nevertheless, it may be employed as a benchmark against which the decisions taken under the models considered here could be assessed. This comparison is carried out by averaging the distance from this optimal value of the average values of players selecting yellow under the experimental results and Nash and Rosenthal (for certain values of parameter *t*) equilibria, which can be calculated by using Fig. 2. From these results, it turns out that the Nash equilibrium is the furthest away from optimality, while the results under the Rosenthal equilibrium at $t=0.05$ produce decisions much closer to the optimal (on the average) than under the Nash equilibrium. The experimental results are also much closer to the optimal decisions than under the Nash equilibrium and slightly closer than under the Rosenthal equilibrium at $t=0.05$. Notice that the (completely) random choice (corresponding also to the Rosenthal equilibrium at $t=0.0$) presents results that are the closest of all previous to the optimal. It should be noted though that the very good closeness of the random to the optimal may be due to the fact that $S=0.5N$ in half of our cases (that is, the optimal decision is by construction that of the random choice policy); for the other half of our cases we have that $S=0.25N$. Separating the two cases, it can be seen that for the case of $S=0.25N$ the random choice is not better than the corresponding results for the Rosenthal equilibrium at $t=0.05$ or the experimental results, while the random choice is still better than the corresponding results under the Nash equilibrium.

All in all, we could say that the (real) players make better (more optimal) decisions than what the Nash equilibrium dictates. Also, the bounded rationality Rosenthal model at t = 0.05 produces decisions that are closer to the optimal than those foreseen by the Nash equilibrium. Results for the Rosenthal equilibrium at $\hbox {t}=0.02$ (more random than for $\hbox {t}=0.05$) gave a better match to the experimental results for the ‘hardest’ to decide cases (when both the cost of not competing ($Q_{\mathrm{exp}}$) and the cost of competing and failing ($Q_{\mathrm{add}}$) are high). Therefore, it is possible that when both such costs are high it is harder for people to decide (or figure out which decision would be the best) and tend to finally make a decision closer to a random one than in the less ‘hard’ cases.

### Reinforcement learning model

As an alternative explanation of our results, we have attempted to reproduce the experiment with an agent-based model where agents evolve according to the reinforcement learning paradigms (see Ref.^30^; we have followed closely the presentation in Ref.^33^). It is interesting to note that reinforcement learning has already proven quite successful in understanding the behavior observed in experiments (see, e.g.^34–36^). In our case, we have studied a system of 20 agents where each one is characterized by its payoff, aspiration (the minimum payoff the agent would like to receive) and probability to choose the cheap parking. For every round, every agent chooses its action and then it is determined which agents go to each parking. The assignment is done according to choices if possible and randomly if more agents choose the yellow parking lot than there are available spots. Then the agents collect the payoff and their probability to choose yellow is updated. Following Ref.^33^, the dynamics of this probability behaves as follows:

6 $$\begin{aligned} p_{a,t+1}= \left\{ \begin{array}{lll} p_{a,t}+(1-p_{a,t})ls_{a,t} &{} if &{} s_{a,t}\ge 0 \\ \\ p_{a,t}+p_{a,t}ls_{a,t} &{} if &{} s_{a,t}<0 \\ \end{array} \right. \end{aligned}$$

where *a* is the action whose probability is being updated, in our case, choosing the yellow or the blue parking lots. There are three free parameters in this model: first, the initial probability of choosing cheap. To set this value, we have considered an empirical point of view. The initial probability of choosing cheap should be related to the number of seats in the parking; therefore, we are assuming that players know with some accuracy the number of people in the population of parking users, which they knew in the experiment and can be learned in a real situation using the experience. In order to introduce the cost of the parking in the initial process, we consider that agents avert risk proportionally to the difference in the cost between choosing expensive ($Q_{exp}$) and choosing cheap and failing ($Q_{cheap}+Q_{add}$). In order to obtain the correct dimensions, this has to be divided by the endowment. The pattern in the results shows that this second factor should modify the first one. Summing up, we can assume that the probability of choosing cheap initially is:

7 $$\begin{aligned} p_{cheap,0}=S/N\left( 1+\dfrac{Q_{\mathrm{exp}}-Q_{\mathrm{add}}-Q_{\mathrm{cheap}}}{M} \right) , \end{aligned}$$

where *M* is the total amount received per round by a player, and *S*/*N* is the proportion between the number of free spaces in the cheap parking (*S*) and the size of the population (*N*).

The second parameter to set is the one corresponding to the initial aspirations. Aspirations are the threshold that classify the stimulus we obtain from an outcome. If the outcome is above the aspirations, the stimulus is positive and reinforces the action; if it is below, it discourages. The distance between the outcome and the aspiration is a measure of the strength of the stimulus. As there is no information about the perceptions of the outcomes, we consider here a gaussian distribution of the aspirations, as the classification of the stimulus and their strength may depend on several factors including personal circumstances. The mean of this gaussian distribution is lower than $M-Q_{exp}$, considering that a great part of the population will consider positive to park in any parking. However, we consider also ambitious agents, which see as negative to spend on the expensive parking, and only receive positive stimulus when they achieve a spot in the cheap one. In order to model this, we introduce a standard deviation such that an 83% of the agents are not ambitious, leading to our choice of

8 $$\begin{aligned} \mu =M-Q_{\mathrm{exp}}-M/5,\qquad \sigma =M/5. \end{aligned}$$

These aspirations are fixed during the whole simulation, which makes sense in view of the short length of the experimental sessions. Finally, the third parameter, namely the learning rate, which measures how much an action changes our probability to choose it again, has been set to $l=1$.

**Figure 4:**
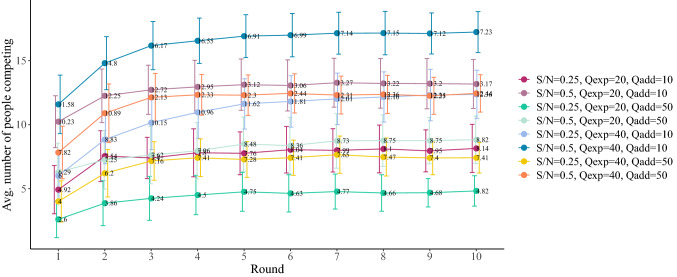
Evolution of simulation averages for each treatment and round. Average number of competitors for each treatment. Error bars represent ± one standard deviation of the mean.

Once we have the initial values, we have run 100 simulations and studied the evolution of the number of competitors, comparing it with the Nash equilibria predicted and the experiment results. Our results are collected in Figs. 2 and 4. As can be seen from the time evolution, our groups take only a few rounds to settle on a behavior, with a constant fraction of them choosing the yellow parking lot. It is interesting to observe that agents in the model reach a more or less constant behavior in a time similar to that found in the experiment (cf. Fig. 2), so this is also well captured by our model. The only discrepancy between model and experiment is the transient process of the first rounds, which seems to be more erratic in the experiment as subjects try different options to get an impression of what is best for them. The final values at the end of the simulation are compared to the results from the experiment with a good agreement as well, and better than the Nash prediction in any event.

If we sum up all the differences in the number of competitors for the cheap places between the experimental results and the reinforcement learning results the sum of these differences is 6.5, which shows a much greater performance than the Nash prediction (sum of differences is 22.23) and a similar performance as the Rosenthal prediction under $\hbox {t}=0.05$ (sum of differences is 4.66). A detailed comparison of the performance induced under the reinforcement learning model and the Rosenthal model would require an extensive parameter space search and larger scale experimentation which is beyond the scope of this paper. Another important result is that not only the average number of agents choosing yellow remains constant after a few rounds, but also 80% of them repeat their decision after the fifth round, with the 50% of them choose the cheaper parking always. This is also in agreement with the experimental observations. In addition, this observation justifies the consideration of a stationary model for capturing the longer-term behavior of users; one such model is the Rosenthal model considered here.

## Discussion

In this paper, we have reported results of an experiment in which we probe on human behavior when people have to choose between a convenient, cheap, but limited parking lot and an unlimited but less convenient one, when choosing the cheap parking lot and failing to find a spot is also costly. Generally speaking, when the number of cheap parking spots is quite limited (1 per every 4 possible users), people tend to collectively reproduce the prediction of the Nash equilibrium; only in one case (when the expensive parking lot is much more expensive and the cost of not finding a spot in the cheap one is low) people behave more cautiously than the Nash prediction. On the other hand, when the cheap parking can accommodate up to 1 of each 2 possible cars, people behave in general again with caution, with the fraction of subjects going for the cheap parking being sizably smaller than the Nash prediction, sometimes by a factor of almost 2.

Given the difficulties of the Nash equilibrium concept to predict the behavior observed in the experiments, we have proposed two alternative explanations for our observation. The first one is a stationary approach based on the idea of the Rosenthal equilibrium^29^. In principle, one could claim that the rationality parameter *t* defining the Rosenthal equilibrium could be dependent on (a) the game itself and (b) the risk/costs involved based on the choices available. Given the game we have and our results, we have found that the parameter $t=0.05$ is a great match to all cases, except for the ‘hardest’ one (the unlimited parking lot is not very expensive but there is a big additional cost for missing the cheap one) for which a better match is that at $t=0.02$ (more random than for $t=0.05$). One possible intuition for this is that when weighing these two options, people find it harder to decide (or figure out which decision would be the best) and tend to finally make a decision closer to random than in the less ‘hard’ cases. This would agree with the remarks made above about the dispersion of individual results when they are close to or far from the Nash equilibrium. Even then, in those simpler situations, the fact that $t=0.05$ is a good match is an indication that there is a relevant degree of randomness in the decision process.

To complete our discussion of the performance (i.e., distances from the experimental results) of the Rosenthal model for rationality parameter t values in $\{0.02, 0.05, 0.01\}$, we note that (a) the Rosenthal model’s predictions are clearly much closer to the experiments than the Nash predictions (distances 22.23 under Nash vs 9.85, 4.66, 8.15) and (b) they are comparable to that under the reinforcement learning model (distance of 6.5). Thus, it looks like a rationality parameter in the range of [0.02, 0.1] seems to capture well the average users’ stationary behavior in this parking (resource) selection problem. The left boundary of that range would be more effective when decisions are harder and, thus, the user behavior tends to be more random, as discussed earlier.

As a complementary approach that is able to incorporate the time evolution of the people decisions, we have studied an agent based model based on reinforcement learning. Our results show a comparable performance with the (stationary) concept of the Rosenthal equilibrium, but most importantly, they support the interpretation we have just discussed by starting from random choices for the initial decision and showing that the group converges to a behavior very similar to the predicted one. Note that in reinforcement learning dynamics, randomness enters prominently as the probability to repeat an action depends strongly on the success or failure of its first use. As getting a spot is random when there is more demand than availability in the cheap parking, the corresponding success or failure of the decision is also partly random. An additional insight provided by our simulations under the reinforcement learning model is that the behavior becomes basically constant after 3 or 4 rounds, not only at the level of the group but also at the level of the individual agent. We thus believe that these dynamics support the use of the Rosenthal equilibrium as a tool to account for bounded rationality, and in turn, suggest that a very natural learning process can explain our observations.

Interestingly, a related approach has been pursued by Guo et al.^37^. They introduce a static game-theoretical model very similar to the one in our experimental design, but different in that their costs are explicitly stated in terms of time spent parking. In such a framework, equilibrium is defined in terms of the fraction of drivers choosing the small parking that makes the time cost of either choice the same. As in the Nash equilibrium approach, this approach only captures the rational aspects of the individual choices. However, the authors devote most of their work to a more elaborated dynamic model, incorporating uncertainty, and fitting their parameters to real data using genetic algorithms. In contrast to our model based on aspirations, the main features of the model in Ref.^37^ are the inclusion of an ambiguity factor and an optimistic index. Their conclusion is in line with ours: the dynamic model turns out to predict much better the occupancy of the parkings they analyze, and the irrational side of decision making (included in the ambiguity factor) appears to constitute an indispensable component of their parking lot choice decision process. We thus see that an independent, different modeling approach combined with data analysis yields a conclusion very similar to our experimental findings and to the conclusions we have drawn from our models.

Our results are attractive in terms of making recommendations for the design of parkings subjected to high demand. Our experiments show that people are able to anticipate better the demand for this type of parkings when the number of available spots is more scarce, while when there is more availability the cheap parking may end up being suboptimally utilized. From this viewpoint, it seems that a good recommendation is to make parking lots with a rather limited amount of spots and to inform the possible users of this prominently. In this context, it may be interesting to consider a complementary approach in terms of a (Stackelberg) game between the authorities and the drivers^38^. The combination of our insights with this optimization approach may prove very useful. On the other hand, we have also seen that people are also more capable to take into account the group behavior when the cost of the alternative parking is not expensive. While further experimentation is needed in order to refine these conclusions, we have provided some guidance that can be useful particularly in the context of decarbonizing the cities by removing traffic from their centers.

Finally, the results in this work are more general than the parking application we have been discussing. What we have seen is that in congestible goods situations, which appear quite often, the Nash equilibrium concept may be unable to predict or help to understand how people behave, particularly in the case where the parameters make it difficult to have educated guesses of what should be the optimal behavior. Importantly, we have not only shown that, but also introduce an alternative approach combining an equilibrium model, namely the Rosenthal one, with a dynamic model based on reinforcement learning that provides an explanation of how that equilibrium is reached. We believe that this approach may be of interest for many other contexts and can prove itself useful to make policy recommendations and to guide decisions in a variety of problems.

## Methods

The experiments were implemented in IBSEN-oTree^39^. The participants played online through a web browser in a computer, tablet or mobile phone. All participants in the experiments signed an informed consent to participate when enrolling in the IBSEN volunteer pool^40^. In agreement with the Spanish Law for Personal Data Protection, their anonymity was always preserved. This procedure was approved by the Ethics Committee of Universidad Carlos III de Madrid, the institution responsible for funding the experiment, and the experiment was subsequently carried out in accordance with the approved guidelines.

We recruited 240 subjects to take part in twelve sessions in June 2019. A total of 36 participants dropped out over the course of the experiment. For those decisions not taken, the software would decide at random for the participant, and these decisions would be marked as “automatic”. There were four types of experimental sessions, which were replicated three times. For the four types of experimental sessions, we tried two *S*/*N* values, two $Q_{\mathrm{exp}}$, and two $Q_{\mathrm{add}}$ values; ensuring that the different Nash equilibria, as computed above for these scenarios, were different and covered the whole solution space. Each participant was randomly assigned to a session. In her session, she participated in four repetitions of the game, for the same *S*/*N* value, and different ordering of the $Q_{\mathrm{exp}}$ and $Q_{\mathrm{add}}$ values, to avoid the ordering bias in the results. This experimental design is summarized in Table 1.

The experimental design results in eight experimental treatments, for each combination of the three experimental parameters: *S*/*N*, $Q_{\mathrm{exp}}$ and $Q_{\mathrm{add}}$, with six replications per treatment. We will refer to each treatment as $EXP\_S/N\_Q_{\mathrm{exp}}\_Q_{\mathrm{add}}$, i. e., $EXP\_0.5\_20\_10$ means $S=10$, $Q_{\mathrm{exp}}=20$, $Q_{\mathrm{add}}=10$.

**Table 1:** Parameterizations of the experimental sessions.

Sessions	S/N	Repetition 1		Repetition 2		Repetition 3		Repetition 4	
		$Q_{\mathrm{exp}}$	$Q_{\mathrm{add}}$	$Q_{\mathrm{exp}}$	$Q_{\mathrm{add}}$	$Q_{\mathrm{exp}}$	$Q_{\mathrm{add}}$	$Q_{\mathrm{exp}}$	$Q_{\mathrm{add}}$
1–3	0.5	40	50	10	50	20	50	40	50
4–6	0.5	20	50	20	50	40	10	20	10
7–9	0.25	40	50	10	50	20	50	40	50
10–12	0.25	20	50	20	50	40	10	20	10

Only participants that made at least 70% of the decisions were considered to have finished the experiment and were accordingly paid. The payment was a participation fee of 5 euros plus their payoff in a randomly selected round converted to euros. The average payment per participant was 13.5 euros.

The experiment was conducted in Spanish. A translated version of the instructions can be found in the “Supplementary information”.

## Supplementary information

Supplementary information.

## Data Availability

Data is available in an structured way at Zenodo public repository with https://doi.org/10.5281/zenodo.3961828.
