# Supplementary material for: Competing for congestible goods: experimental evidence on parking choice
Source: Sci Rep. 2020 Nov 30;10:20803. doi: 10.1038/s41598-020-77711-w (PMC7705686; doi:10.1038/s41598-020-77711-w)
Supplement: Supplementary file 1 — Supplementary information. [file 41598_2020_77711_MOESM1_ESM.pdf]

Competing for congestible goods: experimental evidence on parking choice

María Pereda[1,*], Juan Ozaita[2,3], Ioannis Stavrakakis[4], and Angel Sánchez[2,3,5,6]

[1]UPM
[2]Grupo Interdisciplinar de Sistemas Complejos, Departamento de Matemáticas, Universidad Carlos III de Madrid, 28911 Leganés, Madrid, Spain
[3]Unidad Mixta Interdisciplinar de Comportamiento y Complejidad Social (UMICCS) UC3M-UV-UZ, 28911 Leganés, Madrid, Spain
[4]Department of Informatics and Telecommunications, National and Kapodistrian University of Athens, Greece
[5]Institute UC3M-Santander for Big Data (IBiDat), Universidad Carlos III de Madrid, 28903 Getafe, Madrid, Spain
[6]Instituto de Biocomputación y Física de Sistemas Complejos (BIFI), Universidad de Zaragoza, 50018 Zaragoza, Spain
[*]mariaperedagarcia@gmail.com

Supporting information

Experiment instructions.

The experiment was conducted in Spanish. Here we include a translated version of the instructions.

Initial screen:

"Please wait while the rest of the participants take their seats. "

Second screen:

"The experiment has begun.

On the next page, you will read the instructions for the experiment. These instructions will remain available throughout the experiment.

Please click "Next" when you are ready. "

Instructions screen (same one for the four repetitions, called here "phases"):

"General Instructions

You will participate in an experiment with five phases. At the beginning of each phase you will be able to read its instructions.

Phase F Instructions

You are going to participate in an experiment along with other people. You will be in a group of N people, including yourself.

Imagine that you are a driver who wants to park your car in a car park. You can choose between two types of car park: - "Yellow car park": a public car park lot with a reduced price but limited capacity (there are only S spaces), so you won't always find a place to park. - Blue car park": a private car park lot whose price is higher, but where you will always find a place to park.

You have to choose in which car park you want to park. If you choose the "Yellow Car Park" and there are no spaces left, you will have to go to the "Blue Car Park" afterwards and incur an additional cost to move from the "Yellow Car Park" to the "Blue Car Park".

You will make this decision several times, each decision will be called a "round". At the beginning of each decision, you will be given M points that you can spend to park. The amount you do not spend will be your winnings for each round.

The costs (in points) of each result of your possible decisions are as follows: - If you decide to park in the "Blue Car Park": cost = Qexp - If you decide to park in the "car park Amarillo" and get a space: cost = Qcheap - If you decide to park in the "Yellow Car Park" and do not find a slot to park, so you have to park in the "Blue Car Park": cost of the journey Qadd + cost of the "Blue Car Park" Qexp = total cost.

At the end of the experiment, your earnings in points will be converted into real money. You will be paid for the result of a randomly selected round. At the end of the experiment, the program will randomly select a round and you will be paid for the winnings you made in that round. You are only entitled to be paid if you make at least 70% of the decisions. You will have 30 seconds to make each decision.

For your convenience, these instructions will be available to you throughout the experiment.

Lastly, you will answer 10 simple questions in which you will have to choose between two options, A or B. Subsequently, among the 10 questions, one will be selected at random, which will be used to calculate your earnings in this section. These earnings will be added to the earnings you earned in the previous section.

You will then make your first decision. Press "Next" when you are ready to continue.

"

First decision screen:

"Which car park lot do you want to go to?

- *"Yellow Car Park"*
- *"Blue Car Park"*

(The two options were shown in random order for each participant and round. The instructions were shown at the bottom of the page)."

Waiting screen after each decision:

"Please wait, the other participants are making their decision."

Feedback and decision screen:

"Round r. Your decision.

Your decision in the last round was ... You parked in car park ... Your winnings were P points. L people parked in the Yellow Car Park.

Now you can make a new decision.

Which car park lot do you want to go to?

- *"Yellow Car Park"*
- *"Blue Car Park"*

(The two options were shown in random order for each participant and round. The instructions were shown at the bottom of the page)."

Risk aversion test:

"Lastly, you will answer 10 simple questions in which you will have to choose between two options, A or B. Subsequently, among the 10 questions, one will be selected at random, which will be used to calculate your earnings in this section. These earnings will be added to the earnings you earned in the previous section.

(Table 1 was shown below).

"

Results screen:

"The experiment has ended.

In phases 1 to 4 you will be paid for your results in round r1, in which you scored p1 points. The conversion rate to real money is X points = 1 EUR.

In phase 5 you will be paid for your results in row F, in which you got p2 EUR.

In addition you receive a participation fee of 5 Euros, so your total earnings are T Euros.

Thank you very much for participating."

Table 1. Risk aversion test.

Opción A		Opción B
1 euros if x=1<br>0.8 euros if x>=2	or	0.8 euros if x=1<br>0.05 euros if x>=2
1 euros if x<=2<br>0.8 euros if x>=3	or	0.8 euros if x<=2<br>0.05 euros if x>=3
1 euros if x<=3<br>0.8 euros if x>=4	or	0.8 euros if x<=3<br>0.05 euros if x>=4
1 euros if x<=4<br>0.8 euros if x>=5	or	0.8 euros if x<=4<br>0.05 euros if x>=5
1 euros if x<=5<br>0.8 if x>=6	or	0.8 euros if x<=5<br>0.05 euros if x>=6
1 euros if x<=6<br>0.8 euros if x>=7	or	0.8 euros if x<=6<br>0.05 euros if x>=7
1 euros if x<=7<br>0.8 euros if x>=8	or	0.8 euros if x<=7<br>0.05 euros if x>=8
1 euros if x<=8<br>0.8 euros if x>=9	or	0.8 euros if x<=8<br>0.05 euros if x>=9
1 euros if x<=9<br>0.8 euros if x>=10	or	0.8 euros if x<=9<br>0.05 euros if x>=10
1 euros if x<=10<br>0.8 euros nunca	or	0.8 euros if x<=10<br>0.05 euros nunca

Supplementary figures.

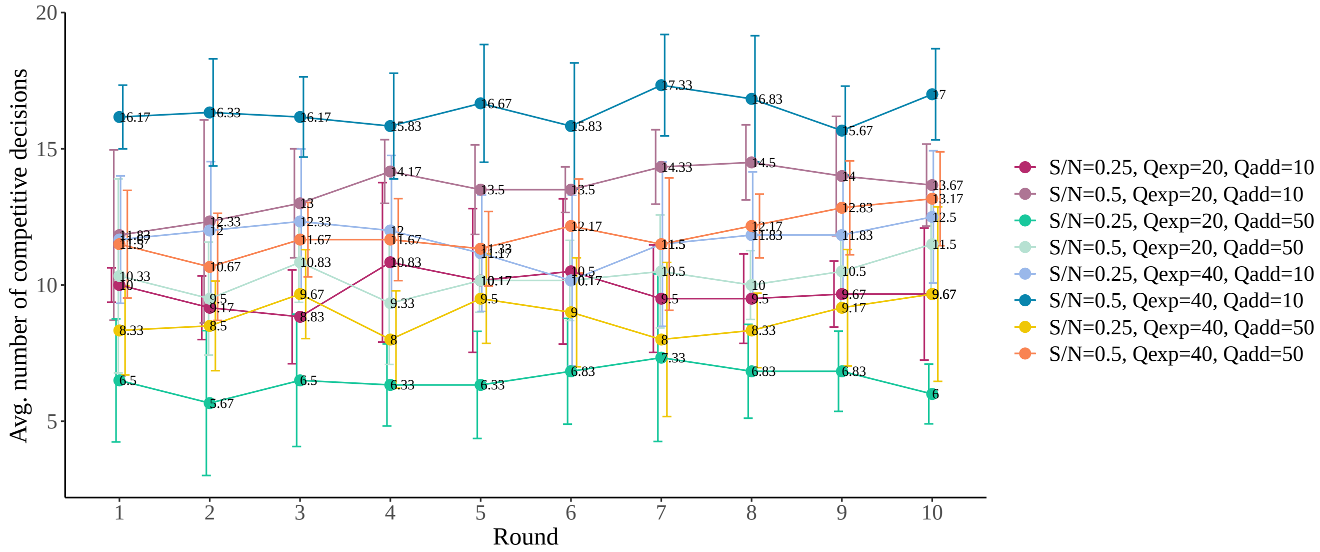

Figure S1. Average number of competitive decisions (people decisions and automatic random decisions, including participants that did not finished the experiment) as a function of round number in the eight treatments of our experiment. Error bars represent ± one standard deviation of the mean.

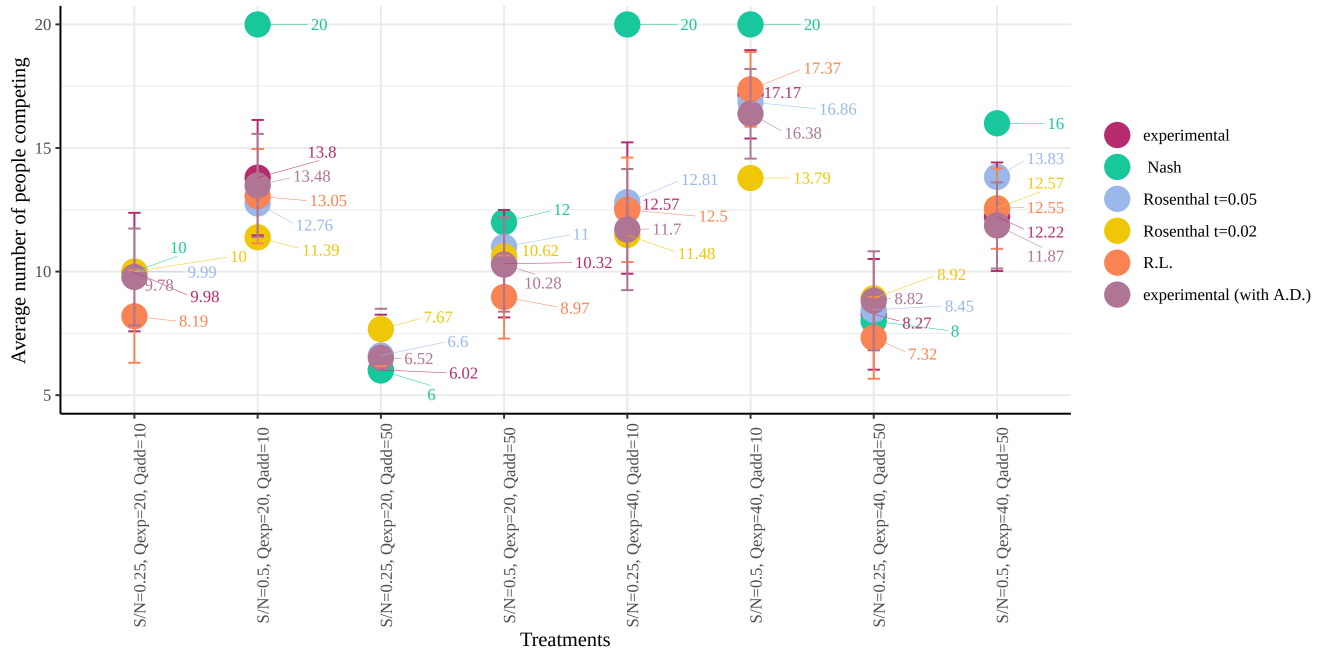

Figure S2. Average number of people competing per treatment and average number of competitive decisions (people decisions and automatic random decisions) versus Nash and Rosenthal equilibria. Magenta: experimental results; turquoise: Nash; blue: Rosenthal, $t = 0.05$; yellow: Rosenthal, $t = 0.02$; orange: Reinforcement learning (R.L.) model; medium pink: experimental results with automatic random decisions and participants that did not finished the experiment. Error bars represent ± one standard deviation of the mean. Points overlap for all sets on the first treatment, except for R.L. simulation results.

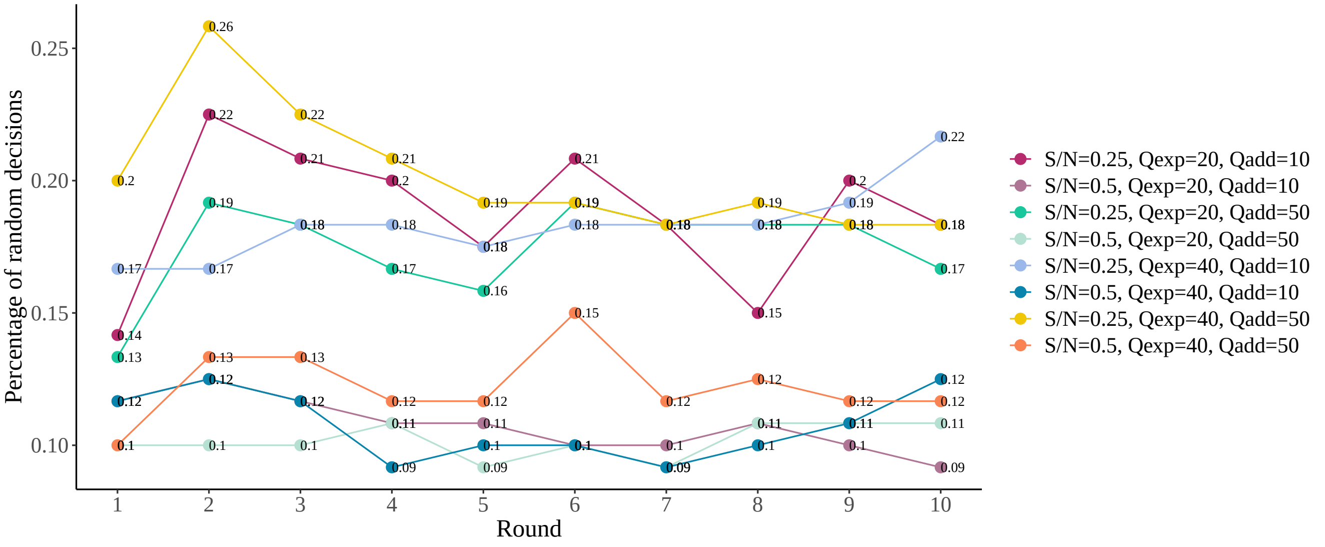

Figure S3. Percentage of random automatic decisions as a function of round number in the eight treatments of our experiment.
